# AAV2/4-*RS1* gene therapy in the retinoschisin knockout mouse model of X-linked retinoschisis

**DOI:** 10.1371/journal.pone.0276298

**Published:** 2022-12-07

**Authors:** Brittni A. Scruggs, Sajag Bhattarai, Megan Helms, Ioana Cherascu, Adisa Salesevic, Elliot Stalter, Joseph Laird, Sheila A. Baker, Arlene V. Drack

**Affiliations:** 1 University of Iowa Institute for Vision Research and the Department of Ophthalmology and Visual Sciences, University of Iowa, Iowa City, Iowa, United States of America; 2 Carver College of Medicine, University of Iowa, Iowa City, Iowa, United States of America; 3 Department of Biochemistry, University of Iowa, Iowa City, Iowa, United States of America; 4 Interdisciplinary Graduate Program in Genetics, University of Iowa, Iowa City, Iowa, United States of America; 5 Department of Pediatrics, University of Iowa, Iowa City, Iowa, United States of America; Iveric Bio, UNITED STATES

## Abstract

**Objective:**

To evaluate efficacy of a novel adeno-associated virus (AAV) vector, AAV2/4-*RS1*, for retinal rescue in the retinoschisin knockout (*Rs1*-KO) mouse model of X-linked retinoschisis (XLRS). Brinzolamide (Azopt^®^), a carbonic anhydrase inhibitor, was tested for its ability to potentiate the effects of AAV2/4-*RS1*.

**Methods:**

AAV2/4-*RS1* with a cytomegalovirus (CMV) promoter (2x10^12^ viral genomes/mL) was delivered to *Rs1*-KO mice via intravitreal (N = 5; 1μL) or subretinal (N = 21; 2μL) injections at postnatal day 60–90. Eleven mice treated with subretinal therapy also received topical Azopt^®^ twice a day. Serial full field electroretinography (ERG) was performed starting at day 50–60 post-injection. Mice were evaluated using a visually guided swim assay (VGSA) in light and dark conditions. The experimental groups were compared to untreated *Rs1*-KO (N = 11), wild-type (N = 12), and *Rs1*-KO mice receiving only Azopt^®^ (N = 5). Immunofluorescence staining was performed to assess RS1 protein expression following treatment.

**Results:**

The ERG b/a ratio was significantly higher in the subretinal plus Azopt^®^ (p<0.0001), subretinal without Azopt^®^ (p = 0.0002), and intravitreal (p = 0.01) treated eyes compared to untreated eyes. There was a highly significant subretinal treatment effect on ERG amplitudes collectively at 7–9 months post-injection (p = 0.0003). Cones showed more effect than rods. The subretinal group showed improved time to platform in the dark VGSA compared to untreated mice (p<0.0001). RS1 protein expression was detected in the outer retina in subretinal treated mice and in the inner retina in intravitreal treated mice.

**Conclusions:**

AAV2/4-*RS1* shows promise for improving retinal phenotype in the *Rs1*-KO mouse model. Subretinal delivery was superior to intravitreal. Topical brinzolamide did not improve efficacy. AAV2/4-*RS1* may be considered as a potential treatment for XLRS patients.

## Introduction

X-linked retinoschisis (XLRS) is an early onset macular dystrophy associated with abnormal or deficient retinoschisin protein (RS1), which is mainly secreted by photoreceptors and is involved in retinal intercellular adhesion and matrix architecture [1, 2]. There are numerous retinoschisin gene (*RS1*) mutations known to lead to the abnormal retinal architecture (intraretinal cysts and schisis cavities) [3]. Affecting approximately 1:5,000 to 1:25,000 live births [3], XLRS causes reduced visual acuity in male infants and children and increases the risk of developing severe, bilateral vision loss from macular atrophy, retinal detachments, and/or amblyopia [4, 5].

Optical coherence tomography (OCT) demonstrates extensive schisis of the inner retinal layers throughout the macula and, in some cases, the retina periphery [6]. Many groups, including our own [7], Apushkin [8], Genead [9], Pennesi [10], and others [11–14], have reported that carbonic anhydrase inhibitor (CAI) treatment (e.g., acetazolamide, dorzolamide, brinzolamide) improves foveoschisis and visual acuity in XLRS patients. Although helpful in select patients, topical CAIs cannot fully treat XLRS; instead, curative treatment would require RS1 replacement. A Phase I/IIa trial has been performed to investigate human gene therapy for XLRS [15]; the three-dose-escalation safety study over 18 months demonstrated overall tolerability to a single intravitreal injection of adeno-associated virus (AAV) 8-*RS1* vector [15], which had originally shown promise in the retinoschisin knockout mouse (*Rs1*-KO) [16]. In the human trial, anatomic improvement was noted in one patient; however, higher vector titer correlated with increased intraocular inflammation, including vitritis and one case of vasculitis [15].

Wiley, et al. evaluated the transduction efficiency and tropism of seven different AAV2 serotypes on human retinal explants, and AAV2/4 was found to be particularly efficient at transducing multiple cell types, including photoreceptor cells [17]. In this assay, AAV2/8 produced the lowest transduction efficiency in human photoreceptor cells despite several other studies demonstrating that AAV2/8 robustly transduces the outer retina of mice [16, 17]. Building on the previous studies in the *Rs1* mouse, our research team tested the hypotheses that subretinal and intravitreal injections of a novel vector, AAV2/4-*RS1*, could correct the retinal phenotype in the *Rs1*-KO mouse detectable by electroretinography (ERG), OCT, immunofluorescence, and Visually Guided Swim Assay (VGSA). This study also evaluated whether topical brinzolamide (Azopt^®^) potentiates the effects of AAV2/4-*RS1* therapy.

## Materials and methods

### Animals

All animal procedures were approved by the Institutional Animal Care and Use Committee (IACUC) of the University of Iowa and conducted in accordance with the ARVO Statement for the Use of Animals in Ophthalmic and Vision Research. Two female *Rs1*-KO/+ C57Bl/6J mice were generously provided by Dr. Paul Sieving at the National Eye Institute. The *Rs1*-KO mouse model was generated as described previously [18]. Two wild-type (WT) C57Bl/6J male mice were obtained from Jackson Laboratory for breeding pairs, and all experimental mice were descended from these breeding pairs. In total, there were eight mouse groups (N = 54 mice): two untreated WT, five subretinal AAV2/4-*RS1*-treated WT, five intravitreal AAV2/4-*RS1*-treated WT, 11 untreated *Rs1*-KO (KO), five topical Azopt^®^-treated *Rs1*-KO (Azopt^®^), 11 subretinal AAV2/4-*RS1* plus topical Azopt^®^-treated *Rs1*-KO (Subretinal + Azopt^®^), 10 subretinal AAV2/4-*RS1*-treated *Rs1*-KO (Subretinal), and five intravitreal AAV2/4-*RS1*-treated *Rs1*-KO (Intravitreal). Male hemizygous mice were used in this study to replicate the disease in humans.

For all treated mice, one eye was injected and/or treated topically, while the fellow eyes remained untreated as controls. For groups receiving Azopt^®^ (1% brinzolamide ophthalmic suspension; Novartis Pharmaceuticals Corporation, Morris Plains, NJ), a single drop was administered topically twice daily starting immediately post-injection and ending at the first post-treatment ERG date. The mouse was scruffed with one hand while one drop of Azopt^®^ was placed on top of the treatment eye with that eye facing upward. The mouse was held in this position for 30 seconds, then the excess liquid was wicked away from the eye with a Kimwipe (Kimtech, Kimberley-Clark, Roswell, GA).

### Adeno-associated virus (AAV) 2/4-*RS1* and AAV2/4-enhanced green fluorescent protein (*eGFP*) vectors

Recombinant AAV2/4 vectors expressing either enhanced green fluorescent protein (*eGFP*) or *RS1* under control of the cytomegalovirus (CMV) promoter were generated in the laboratory of Dr. Budd A. Tucker as previously described [19, 20] and provided as a gift for use in the studies presented (S1 Fig).

### Intravitreal and subretinal injections of AAV2/4-*RS1* in a mouse model

Mice were treated at postnatal day 60–90. Body temperature was maintained at a constant temperature of 38°C using a heating pad. Subretinal injection was performed using sterile technique under an OPMI Lumera 700 surgical operating microscope (Carl Zeiss, Meditec AG, Germany) as described previously [21]. Mice were anesthetized using a ketamine/xylazine mix (0.1 mL/20 g weight at a concentration of 17.5 mg/mL ketamine and 2.5 mg/mL xylazine), and topical tropicamide 1% (Akron Inc, Lake Forest, IL) eyedrops were applied for pupillary dilation. Once under the microscope, topical povidone iodine 10% solution, and proparacaine 1% (one drop each) were applied. A limbal conjunctival peritomy was made superotemporally using 0.12 forceps and Vannas scissors (Bausch and Lomb/Storz Ophthalmics, Rochester, NY).

For subretinal injections, a sclerotomy was made just posterior to the limbus using a 30-gauge half-inch needle at a 45-degree angle viewed through the operating microscope. A 2μL injection (4x10^9^ viral genomes) was then delivered through this scleral opening by inserting a 33-gauge blunt needle on a Hamilton syringe at about a 60-degree angle to the sclera (Hamilton Company, Reno, Nevada) inserting it between sclera and retina, and a second investigator depressed the plunger. The needle was left in place for several seconds after the fluid was delivered. The resulting retinal bleb could be visualized through the operating microscope. If no bleb formed, or if extensive hemorrhage was seen, the eye was excluded. This technique for subretinal bleb formation in the mouse leads to blebs of different sizes in different animals. The blebs are scored based on appearance through the operating microscope as small, moderate, or excellent with excellent blebs encompassing ≥ 50% of the retina and small blebs less than 25%. Excellent blebs often encircled the optic nerve, whereas moderate were more localized to one side of the nerve. Eyes with small blebs were excluded and data is not present in this study.

For intravitreal injection, 1μL (2x10^9^ viral genomes) was delivered through the sclera posterior to the lens edge at a 90-degree angle using a sharp 33-gauge needle on a Hamilton syringe. If extensive vitreous blood was noted, or if a large subretinal bleb was identified, the eye was excluded. The mouse crystalline lens is disproportionately large compared to human lenses; therefore, careful injection techniques are critically important [21, 22]. The lens was inspected through the microscope after injection to exclude eyes with visible lens rupture. The ideal depth of needle penetration was determined prior to these experiments and was marked on the needle to assist with proper depth.

### Light and dark-adapted electroretinography (ERG)

ERG was obtained using the Celeris system from Diagnosys (Diagnosys LLC, MA, USA) at six weeks (50–60 days) after treatment initiation and again at 7–9 months after treatment. After overnight dark-adaptation, mice were anesthetized with an intraperitoneal injection of ketamine/xylazine mixture (0.1mL/20g weight at a concentration of 17.5mg/mL ketamine and 2.5mg/mL xylazine). ERGs were recorded simultaneously from the corneal surface of each eye after pupil dilation using 1% tropicamide (Akron inc, Lake Forest, IL) using the electrode/stimulator combination (Diagnosys), which also served as reference and ground. The low cutoff was 0.125 Hz and high cut off was 33 Hz with a gain set to 8. A drop of 0.3% Hypromellose (Genteal gel, Alcon Laboratories, Fort Worth, TX.) was placed on the corneal surface to ensure electrical contact and to maintain corneal integrity. Body temperature was maintained at a constant temperature of 38°C using the system heat pad. Dim red light was used for illumination until dark-adapted testing was completed. A dark-adapted latency intensity ERG protocol was used, with intensities ranging from 0.003 cd.s/m^2^ to 100.0 cd.s/m^2^. Following the latency intensity protocol, the mice were light-adapted (LA) for 10 minutes then the LA 3.0 flash and 5 Hz flicker cone testing was performed. ERG a-wave and b-wave data were collated in Microsoft Excel and analyzed using GraphPad Prism software (GraphPad Software Inc. San Diego, CA).

### Fundus photography and optical coherence tomography (OCT)

Fundus photographs were obtained using a Micron III camera system (Phoenix Technology group, Pleasanton, CA). Images were processed using Photoshop (Adobe, San Jose, CA). OCT was performed using the spectral domain (SD) Envisu Image Guided SD-OCT system. Bioptigen InVivoVue software (Leica Biosystems Inc, Buffalo Grove, IL) allowed segmentation and thickness estimation for all retinal layers, including: nerve fiber layer (NFL) with ganglion cell layer (GCL), inner plexiform layer (IPL), inner nuclear layer (INL), outer plexiform layer (OPL), outer nuclear layer (ONL), inner segment and outer segment junction (IS/OS), and retinal pigment epithelium (RPE) with choroid.

### Light and dark-adapted visually guided swim assays (VGSA)

At six months of age, untreated WT mice (N = 3) and *Rs1*-KO mice (N = 29) were trained to swim under ambient room lighting (luminance 13.35 cd/m^2^) and under dim red lighting (luminance 4.17E-03 cd/m^2^) in a 42-inch diameter pool (Splash Time, General Foam Plastics Corp, Virginia Beach, VA) to a high contrast visible escape platform [23–25].

A flag was employed on top of the platform to aid in visually guided escape (S1 Video). For photopic testing, mice were trained to find the platform for four days and tested for four days. Immediately following photopic testing, mice were trained for two days on scotopic testing, and tested for four days. Five trials were conducted per mouse per testing day with the platform moved to a random position each trial. Eight platform positions were used throughout the training and testing. The average time to platform (TTP) for each mouse was calculated using the average TTP from the 20 trials over the four consecutive days of testing for each separate lighting condition. Data were collated in Excel spreadsheet and analyzed using GraphPad prism software (GraphPad Software, San Diego, CA).

### Tissue processing and histological procedure

Mice were euthanized by CO_2_ asphyxiation followed by cervical dislocation. Eyes were enucleated and immersed in 4% Paraformaldehyde cold fixative for >4 hours and then stored in phosphate buffered saline (PBS) for later processing. Eyes were dissected, and the anterior segment and the lens were removed. Eyes were embedded in acrylamide solution and then, while maintaining orientation, frozen in optimal cutting temperature compound. Posterior poles were oriented to obtain superior-to-inferior cross sections of the eye cup. Tissue was sectioned at 8μm on a cryostat (Leica Biosystems Inc, Buffalo Grove, IL). Sections were stored at -80°C until immunohistochemical labeling was performed.

### Immunofluorescence of mouse retina

Cryosections were blocked with either 2% bovine serum albumin (BSA) in PBS or 20% goat serum for 30 minutes. Antibody probed sections were labeled with anti-retinoschisin (Anti-RS1) antibody, clone 3R10 (EMD Millipore, Burlington, MA) diluted at 1:50 for 1 hour. Control sections were incubated with PBS alone without primary antibody. All samples were rinsed in PBS and labeled with Alexa Fluor 568 conjugated secondary antibody (Life Technologies Corporation, Carlsbad, CA) diluted at 1:200 in PBS with DAPI (1:5000). All sections were from retina adjacent to the optic nerve; there were two primary antibody probed sections and one control section for every eye analyzed with immunohistochemistry. Histological sections were photographed at 20X resolution using Spot RT3 camera (Diagnostic instruments, Sterling Heights, MI). Two WT mice, two KO, two subretinal-treated KO mice, and two intravitreal-treated KO mice had eyes prepared for immunofluorescence.

### Statistical analyses

Statistical analyses were conducted using a two-tailed two-sample paired t-test to compare b-wave amplitudes of treated and untreated eyes at each luminance in the latency intensity protocol. To compare ERG amplitudes for treated eyes compared to untreated eyes, a linear mixed effects model was used. This analysis used all ERG amplitudes obtained at the latest timepoint (7–9 months post-injection) and took into account that each KO mouse had 10 different measurements (data from two eyes including 3 points on the latency intensity ERG protocol: dark adapted 0.01, dark adapted 0.1, dark adapted 1.0, and two values from the light adapted ERG, the 3.0, and 5 Hz flicker). Statistical analysis of three or more groups was performed using one-way analysis of variance (ANOVA) followed by pairwise comparisons of the mouse groups using *post-hoc* testing with Tukey correction. For comparison of ERG where the b/a ratios obtained from waveforms at various luminances for the untreated and treated eyes were compared, two-way ANOVA (factors: treatment and luminance) with repeated measures and subsequent Sidak’s *post-hoc* testing was performed. Significance for the overall treatment effect and individual pairwise comparisons was defined as p<0.05. One- and two-way ANOVA tests were performed using GraphPad Prism 4.0b for Macintosh (GraphPad Software, San Diego, CA), and all values were reported as mean ± standard error of the mean (SEM). The treatment effect for the swim assay was reported as the mean square for each main effect and the interaction effect divided by the variance (i.e., F value); the degrees of freedom and the total number of trials analyzed were included. GraphPad Prism 4.0b for Macintosh and Photoshop (Adobe, San Jose, CA) were used to generate figures.

## Results

### Post-injection localization of subretinal bleb

AAV2/4-*eGFP* was administered to the subretinal space of WT mice with subsequent fundus photography and OCT testing. No vector-associated toxicities (inflammation, subretinal precipitates, reduction in ERG) were noted in any of the treated WT mice. S2A–S2C Fig demonstrates retinal transduction by AAV2/4-*eGFP* 10 days after subretinal injection. The merged image (S2A Fig) shows a large area of eGFP positivity in the region of the subretinal injection bleb. S2D Fig shows the OCT images of the area adjacent to an intravitreal injection; a trace amount of subretinal fluid near a self-sealing retinal hole formed from the needle track is also present in this eye. S2E Fig shows a representative OCT image taken immediately after subretinal injection. This image demonstrates a small bleb; blebs were scored on a range of very small to moderate to large with large being 50% or more of the retina detached. Eyes with absent or very small blebs, such as the trace amount of subretinal fluid shown following an intravitreal injection, were excluded from analysis. The subretinal fluid seen in the area of the subretinal bleb resolved within 24 hours in most cases.

**Fig 1:**
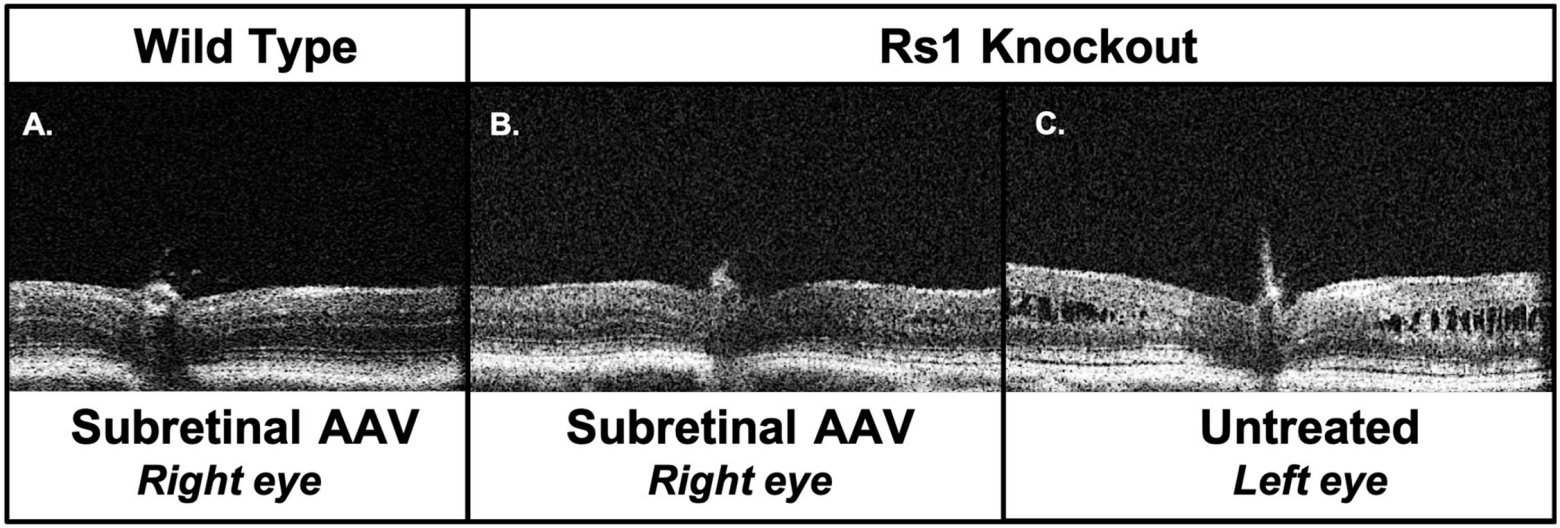
Optical coherence tomography (OCT) demonstrates resolution of intraretinal cysts two weeks after treatment in the *Rs1*-KO mouse. Representative OCT images of a subretinal AAV2/4-*RS1*-treated WT mouse eye (**A**), a treated *Rs1-KO* mouse eye (**B**), and the fellow untreated eye of the same *Rs1-KO* mouse (**C**) two weeks after subretinal AAV2/4-*RS1* with CMV promoter. Mice were P74 at the time of OCT. There was no sign of toxicity in WT. By two weeks after subretinal treatment, the cysts in the treated eye had resolved (**B**) with lamination similar to WT, but cysts persisted in the untreated eye (**C**). The treated *Rs1-KO* mouse had a diffuse bleb in the treated eye. AAV, adeno-associated virus; CMV, cytomegalovirus; KO, knockout; OCT, optical coherence tomography; RS1, retinoschisin; WT, wild-type.

In the next set of experiments, we evaluated the effects of AAV2/4-*RS1* gene therapy. OCT images were taken of retina adjacent to the optic nerve (Fig 1). Comparing the OCT images of the WT and *Rs1*-KO mice, the KO had diffuse intraretinal cysts throughout the INL. Both eyes of *Rs1-KO* mice showed inner retinal cysts around the optic nerves, whereas the WT mice had no cysts. After subretinal gene therapy (P60-90), the cysts improved compared to the fellow untreated eye by two weeks post-injection for most mice; representative images are shown in Fig 1. All untreated eyes had significant number of cysts present at this timepoint. Resolution of cysts on both sides of the nerve was not always seen in all treated eyes and was directly dependent on location of the bleb. *Rs1*-KO mice eventually lose retinal lamination detail with collapse of their intraretinal cysts over time; Zeng, et al. found that there is a significant decline in cavity size between four and eight months for this mouse model [26]. This may correspond to retinal degeneration. Our team chose to not perform serial OCT comparisons in *Rs1*-KO mice older than 5 months as it is difficult to ascertain treatment-related improvements.

### Treatment effects on visually guided swim assay (VGSA) performance

**Fig 2:**
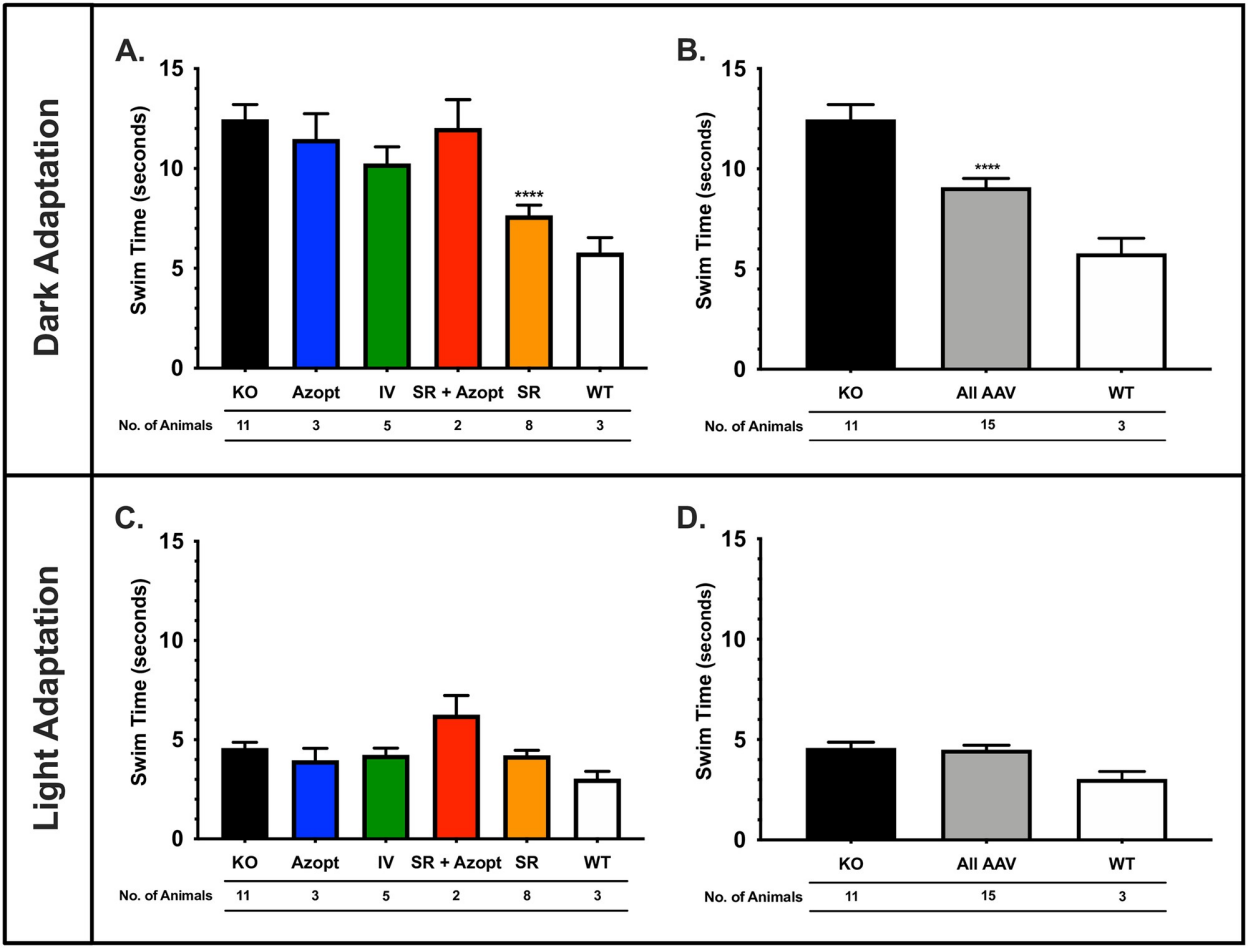
Visually guided swim assays (VGSA) demonstrate better functional vision in the dark in gene therapy treated mice. **(A)** *Rs1*-KO Untreated (KO), *Rs1-KO* treated with topical Azopt^®^ alone (Azopt^®^), intravitreal (IV), subretinal AAV2/4-*RS1* plus topical Azopt^®^ (SR + Azopt^®^), subretinal AAV2/4-*RS1* (SR), and untreated wild-type (WT) mice at postnatal month 6 (4 months after treatment) performed the VGSA in the dark. Better performance, noted as a shorter time to platform (TTP), was seen in the SR group compared to the untreated KO mouse (p<0.0001). The Azopt^®^ (p = 0.0065), IV (p = 0.0277), and SR + Azopt^®^ (p = 0.0099) groups had significantly slower performances than the WT, while the SR group was not different than WT (p = 0.7360). (**B**) When considered together, the groups treated with intravitreal or subretinal AAV2/4-*RS1* with or without topical Azopt^®^ had better TTP compared to untreated *Rs1-KO* (p<0.0001) but slower times compared to WT mice in the dark swim assay (p = 0.0241). (**C**) The same comparisons as (**A**) were performed for mouse performance during the light adapted VGSA; there were no significant differences between groups in the light. (**D)** When considered together, the treated group was not different than the WT in the light. One-way ANOVA with post-hoc testing was used to compare all groups with significance defined as ****, p<0.0001. The number of mice per group is provided. AAV, adeno-associated virus; ANOVA, analysis of variance; IV, intravitreal; KO, knockout; RS1, retinoschisin; SR, subretinal; VGSA, visually guided swim assay; WT, wild-type.

Mice (N = 32) underwent training for swim assays in both the light and the dark four months post treatment. Comparing time to platform (TTP) in the dark for untreated KO (N = 11), Azopt^®^-treated (N = 3), intravitreal treated (N = 5), subretinal + Azopt^®^ treated (N = 2), subretinal treated (N = 8), and WT (N = 3) mice in the VGSA, there was a significant difference in performance based on treatment received (Fig 2A and 2B). In the dark, the subretinal group had significantly faster TTP than the untreated KO mice (p<0.0001) (Fig 2A, S1 Video). No other experimental groups had significantly better TTP compared to untreated KO mice (Fig 2A). All *Rs1*-KO groups had significantly slower TTP compared to WT in the dark, as expected (S1 Video). There was no difference between any of the mouse groups when performing the VGSA in the light, including no difference between treated or untreated *Rs*1-KO and WT (Fig 2C and 2D). This was due to normal TTP in the light in untreated *Rs1*-KO mice at 4 months of age.

### Evaluation of retinal function using ERG

**Fig 3:**
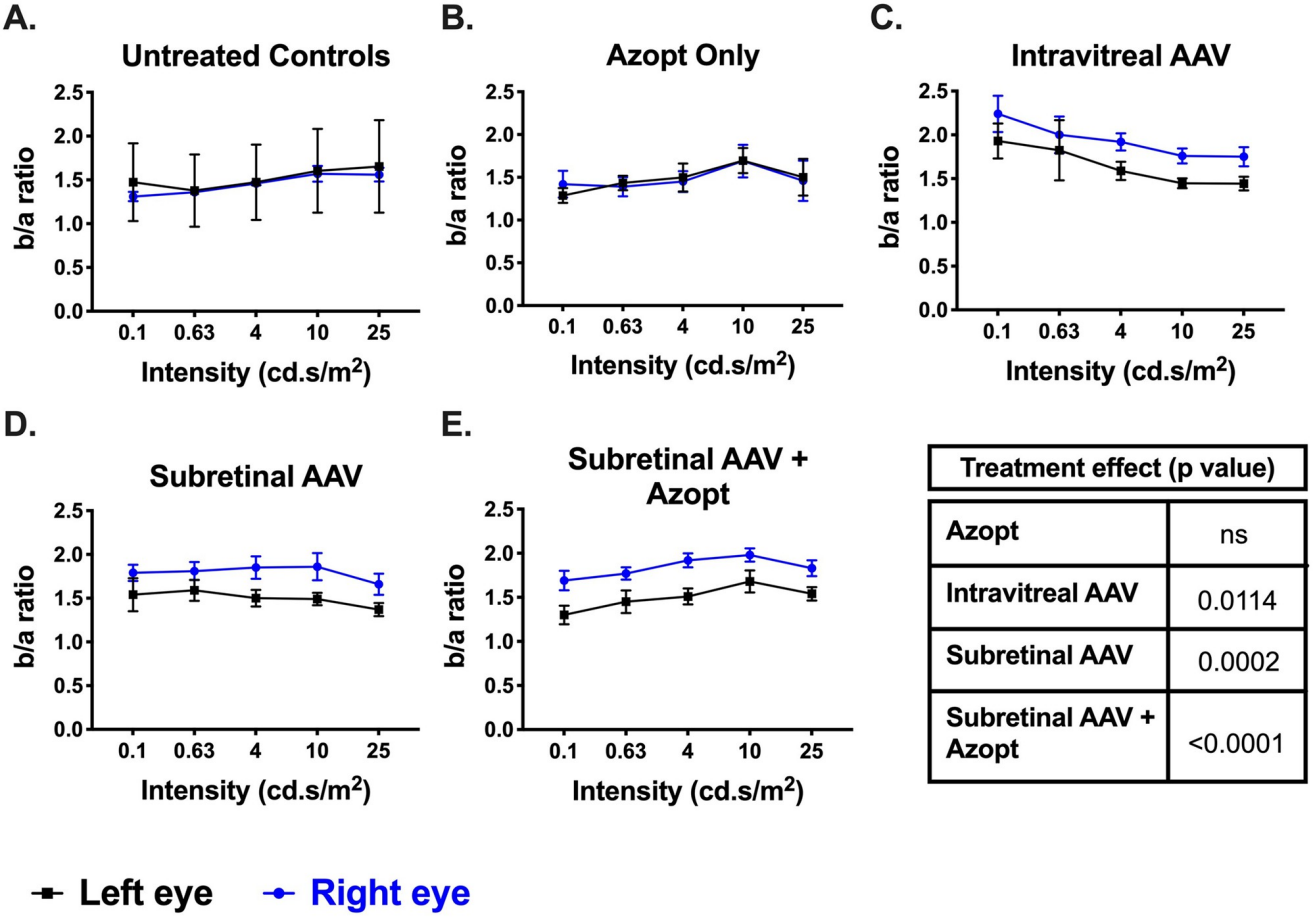
Gene therapy improves b/a ratio on full-field ERG. **(A-E**) Dark adapted full-field ERG testing at various luminances was performed at postnatal month 3.5 (50–60 days after treatment initiation) for untreated and treated *Rs1-KO* mouse eyes, including: untreated *Rs1-KO* controls (**A**, N = 3), topical Azopt^®^ alone (**B**, N = 3), intravitreal AAV2/4-*RS1* (**C**, N = 5), subretinal AAV2/4-*RS1* (**D**, N = 10), and subretinal AAV2/4-*RS1* plus topical Azopt^®^ (**E**, N = 11). The b to a-wave ratios were measured for all eyes at each luminance then averaged per group. Untreated eyes are shown in black across all mouse groups, whereas treated eyes are shown in blue for all experimental groups (**B-E**). Both eyes of the untreated KO group (**A**) received no therapy. The Azopt^®^ group (**B**) showed no treatment effect, whereas the intravitreal group (**C**) and both subretinal groups (**D & E**) had improved ERG recordings in the treated eyes compared to untreated eyes. Two-way ANOVA with post-hoc testing was used to determine significance of the treatment effect; all treated eyes had significantly better b to a-wave ratios than untreated except for the Azopt^®^ treatment group. P values for the experimental groups are provided. All points represent mean +/- SEM. AAV, adeno-associated virus; ANOVA, analysis of variance; ERG, electroretinography; KO, knockout; RS1, retinoschisin; SEM, standard error of the mean.

ERG recordings allowed monitoring of post-therapy changes as the *Rs1*-KO mouse has profound reduction of the a-wave and b-wave amplitudes, suggesting photoreceptor and synaptic dysfunction, respectively. The b-wave amplitude and b/a ratio were the most sensitive measures of differences between the treatment groups. The b/a ratio has been reported to be a useful marker in XLRS [27, 28]. The b/a ratio was almost identical between fellow eyes in untreated *Rs1*-KO animals (N = 3; Fig 3A) and in the Azopt^®^-only treatment group (N = 3; Fig 3B). There was an increased (i.e., more normal) b/a ratio in the treated eyes of the intravitreal (N = 5; p = 0.0114), subretinal (N = 10; p = 0.002), and subretinal plus Azopt^®^ (N = 11; p<0.0001) groups at 50–60 days post treatment (Fig 3C-3E) suggesting better transmission of the signal from photoreceptor to bipolar cells.

**Fig 4:**
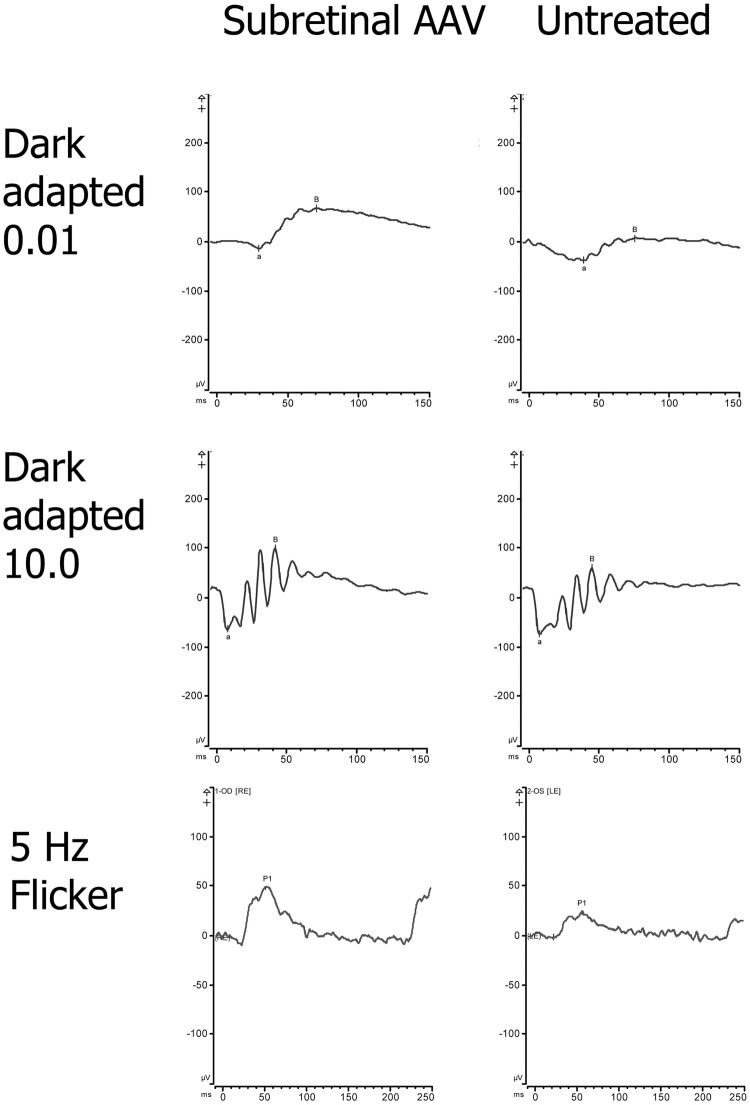
ERG amplitudes are more robust in *Rs1*-KO eyes treated with AAV2/4-*RS1*. Dark-adapted ERG and light-adapted ERG representative waveforms for an *Rs1-KO* mouse with subretinal AAV-treatment in one eye and the fellow eye untreated at postnatal month 5.5 (3.5 months after treatment). For each tracing, time is shown in milliseconds (ms; x-axis) and amplitude is shown in microvolts (μv; y-axis). The b-wave and a-wave amplitude for each waveform is noted for the dark-adapted ERG, amplitude P1 is noted for 5Hz flicker. AAV, adeno-associated virus; ERG, electroretinography; RS1, retinoschisin.

ERG waveforms from a representative *Rs1-KO* mouse 3.5 months post-injection with one eye subretinally treated and the other control can be seen in Fig 4 (Fig 4). ERG amplitudes of an *Rs1-KO* mouse over time with and without subretinal treatment with AAV2/4-*RS1* are shown in S3 Fig compared to ERG waveforms from a WT mouse at similar timepoints.

**Fig 5:**
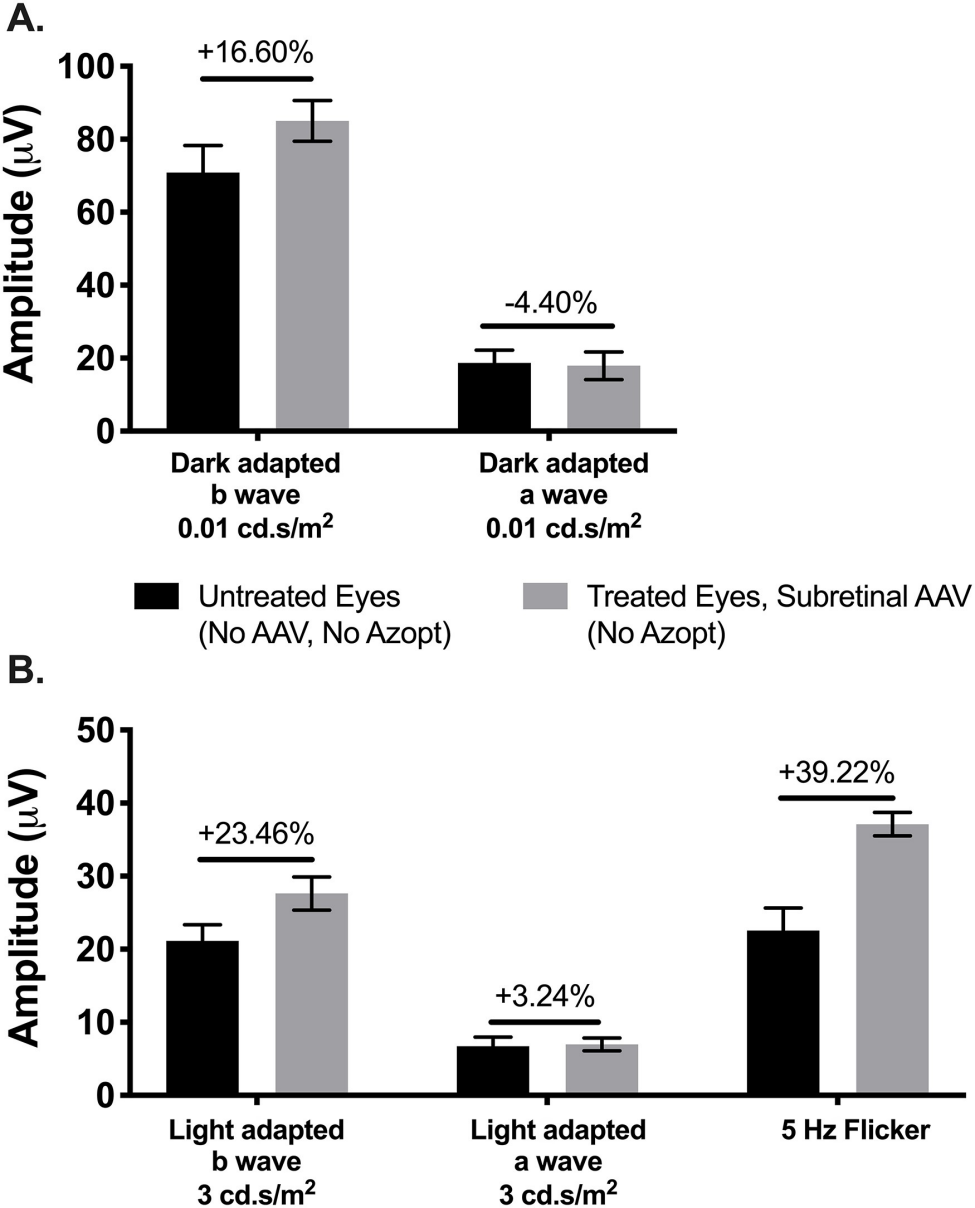
ERG amplitudes are higher in subretinally treated eyes in dark and light conditions. Dark-adapted (**A**) and light-adapted (**B**) ERG were performed at postnatal month 9–11 for *Rs1-KO* mice that either received subretinal AAV-treatment (N = 4; without Azopt^®^) or no treatment (N = 12). The amplitudes in microvolts (μv; y-axis) were averaged for all treated eyes (grey bars) and compared to all untreated eyes (black bars) for dim flash (0.01 cd.s/m^2^), bright flash (3 cd.s/m^2^), and 5 Hertz (Hz) flicker testing. Similarly, the a-wave amplitudes were compared between untreated and treated eyes. Percentage differences for rod predominant ERG testing (**A**, dark-adapted) and cone predominant ERG testing (**B**, light-adapted and flicker) were calculated for determination of treatment effect. There was a significant difference in b-wave amplitude between treated right eyes and untreated left eyes, but no significant difference between the rescue of rods (**A**) and cones (**B**). AAV, adeno-associated virus; ERG, electroretinography; RS1, retinoschisin.

The b/a ratio will be increased (more normal) whether the b-wave amplitudes (bipolar cells) increase or the a-wave amplitudes (photoreceptor signal) decrease. If the b/a ratio increases due to worsening photoreceptor amplitudes, a “better” b/a ratio may be falsely considered a positive treatment effect, therefore the values of a- and b-waves were analyzed individually as well as the b/a ratio. At 7–9 months post treatment, the a- and b-wave amplitudes for eyes treated with subretinal gene therapy alone (N = 4) were averaged and compared to all untreated eyes (N = 12). As shown in Fig 5A, there was a 16.60% higher b-wave amplitude in subretinal-treated eyes than untreated on dark-adapted dim flash (rod) testing (85.03 vs. 70.91; p = 0.3100) and a 23.45% higher amplitude b-wave amplitude on single bright flash (cone) testing (27.63 vs. 21.15, p = 0.1384). There was minimal change in the a-wave amplitudes for the same testing at this timepoint (Fig 5A and 5B). When collectively comparing light adapted, flicker, and dark-adapted ERG amplitudes of *Rs1-KO* mice at postnatal months 9–10, there was a significant treatment effect with higher b-wave amplitudes (all treated vs. untreated eyes, p = 0.0003; N = 12) without a-wave change (S4 Fig). Thus, it was primarily higher b-wave amplitudes in treated compared to untreated eyes leading to improved b/a ratio.

On light adapted 5 Hz flicker (cone) testing (Fig 5B), the subretinal-treated eyes had a 39.22% higher amplitude than untreated (37.11 vs. 22.56; p = 0.0193, q = 0.0590) The percentage difference between treated and untreated eyes showed a trend for more effect of gene therapy on cone predominant amplitudes (light-adapted and flicker) than for rod predominant amplitudes (dark-adapted), though this was not statistically significantly different (p = 0.8844, two-sample t test).

### Gene therapy increases RS1 protein expression in the mouse retina

**Fig 6:**
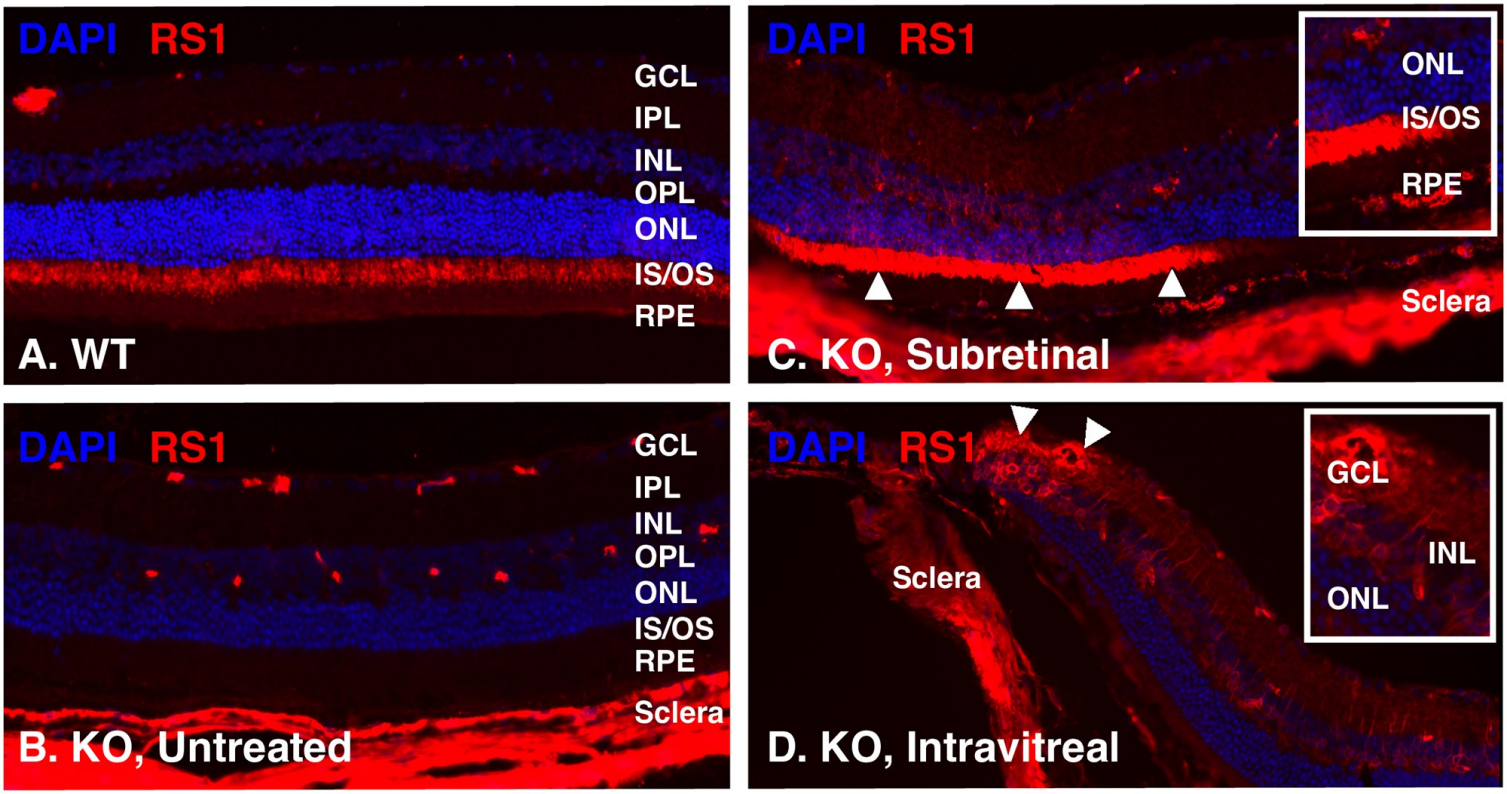
Human RS1 protein expression detected using immunofluorescence following AAV2/4-*RS1* gene therapy. Untreated wild-type (**WT, panel A**), untreated *Rs1-KO* (**KO, panel B**), subretinal treated *Rs1-KO* (**Subretinal, panel C**), and intravitreal treated *Rs1-KO* (**Intravitreal, panel D**) mouse retinas were processed and stained with DAPI (blue) for nucleus visualization and with a primary antibody to RS1 and Alex Fluor 568 conjugated secondary antibody (red). Representative merged images demonstrate normal RS1 expression in the outer retina of WT (**A**) and absence of RS1 expression in the outer retina of KO (**B**). In panel C, localized outer retinal expression is evident in the subretinal treated KO in the area of the treatment bleb (up arrowheads), but not adjacent to it. In panel D, RS1 expression can be seen in the inner retina (down arrowheads) of an intravitreal treated KO eye, but not in the outer retina. The subset images in panels C and D show higher magnification of the area of RS1 transgene transduction. AAV, adeno-associated virus; DAPI, 4′,6-diamidino-2-phenylindole; GCL, ganglion cell layer; INL, inner nuclear layer; IPL, inner plexiform layer; IS/OS, inner segment/outer segment; KO, knockout; ONL, outer nuclear layer; OPL, outer plexiform layer; RPE, retinal pigment epithelium; RS1, retinoschisin; WT, wild-type.

Untreated WT (N = 2), untreated KO (N = 2), intravitreal-treated KO (N = 2), and subretinal-treated KO (N = 2) mouse retinas were evaluated using immunofluorescence for nucleus visualization and presence of RS1 expression; representative images are shown in Fig 6. The merged images show the normal RS1 expression in the outer retina of both the WT (Fig 6A) and subretinal-treated mice (Fig 6C) at the level of the photoreceptor inner segment/outer segment (IS/OS) junction. As expected, the untreated *Rs1*-KO mice (Fig 6B) had no evidence of RS1 staining. When comparing the subretinal and intravitreal groups, the RS1 expression was seen in different layers with the inner retina transduced only by the intravitreal gene therapy (Fig 6D) and the outer retina transduced only by the subretinal gene therapy (Fig 6C).

## Discussion

Human gene therapy for XLRS has shown promise using intravitreal injection of adeno-associated virus AAV8-*RS1* vector [15], which was originally tested in the retinoschisin knockout mouse model [16]. The 2018 safety and tolerability clinical trial demonstrated that intravitreal injection of the AAV8-*RS1* vector was tolerated in most participants, although dose escalation to 10^11^ vector genomes per eye led to inflammation requiring topical and oral corticosteroids [15]. Such inflammation associated with increased number of injected vector genomes warrants additional studies to optimize the vector, administration route, adjunctive therapy, and/or immunosuppression regimens.

Compared to all AAV2 serotypes, the AAV2/4 vector has been shown to have the highest transfection efficiency within both the outer and inner retina for human retinal explants [17]. In contrast, few human photoreceptors in human retinal explants achieved transduction with the AAV2 and AAV8 vectors [17], which are the only vectors [15, 29] tested in XLRS clinical trials to date (NCT02416622, AAV2tYF; NCT02317887, AAV8). The promise of better efficacy using a novel vector guided this pre-clinical study. Specifically, this study tested intravitreal and subretinal routes of administering a novel AAV2/4-*RS1* vector using the CMV promoter for increased transduction efficacy of the outer retina, where RS1 is generated endogenously as a soluble protein.

There are several similarities and differences that should be noted between the current study and that of the AAV8-*RS1* study [16] using the same knockout mouse model. First, the number of vector genomes injected per eye (2E9-4E9) was comparable in the current study to the highest dose administered by Bush, *et al* [16]. Second, the mice in our study were older by 1–3 months at the time of injection, potentially leading to less robust rescue, but better mimicking the situation in human patients who are unlikely to be treated in infancy or early childhood. Third, there were fewer mice per group in our study, which may affect statistical analysis.

Mimicking severe XLRS mutations in patients, the knockout mouse model used in this study has no functional RS1 protein [18, 30]. For both mice and humans, loss of RS1 leads to abnormal retinal architecture and synaptic dysfunction [2, 6, 10, 24, 27]. This reduced communication between photoreceptors and bipolar cells results in the characteristic electronegative ERG with a reduced b-wave compared to the a-wave [4]. Regardless of administration route, AAV2/4-*RS1* improved ERG b to a-wave ratios compared to untreated eyes. The improved ratios were likely related to better bipolar cell to photoreceptor connectivity (increased b-wave amplitudes) in treated eyes. Given the a-wave amplitudes were relatively constant, it is unlikely that photoreceptor loss related to mechanical effects from subretinal bleb formation played an important role.

Intravitreal injections, although less traumatic, demonstrated less effect on the b-wave in our study, likely due to expression only in the inner retina. Retinoschisin is produced in photoreceptors, and with AAV2/4-*RS1* delivered intravitreally, we did not see expression in the photoreceptor layer. Although secretion of RS1 from inner retina could be partially therapeutic due to protein transport across the retina, Byrne et al. previously demonstrated that the greatest long-term rescue in the *Rs1*-KO mouse was observed when *photoreceptors*, not inner retina, produce retinoschisin [31]. This is in agreement with the results herein; the improved outer retina transduction of the subretinal group led to the greatest ERG improvements and, more importantly, excellent outcomes in the visually guided swim assay.

The most remarkable initial improvement in ERG was improved b to a-wave ratio 3.5 months after treatment, suggesting a beneficial effect on bipolar and inner retina/amacrine cells. By 7–9 months after treatment, both treated and untreated eyes had improved oscillatory potentials, suggesting a disease course independent of treatment in the bipolar cells and inner retina, which has been previously described in this mouse model [30]. However, there was a significant treatment effect when looking at collective (light-adapted and dark-adapted) ERG data for 9 to 11-month-old mice (7–9 months after subretinal injection). The single flash and 5 Hz flicker trended better in the treated eyes at this later timepoint. This possibly suggests that the disease progression may be different in cones vs rods, and the cone cells may be more amenable to rescue. This cone cell response would be expected to correlate with long-term central acuity in humans. Given late macular atrophy and central scotoma are the most serious long-term sequelae in humans, gene therapy may have a role in delaying or abolishing this progression.

Most XLRS patients have schisis cavities as the predominant clinical finding, yet *Rs1*-KO mouse demonstrate decrease in cyst volume after four to six months of life with concomitant thinning of the retina and widespread loss of crisp retinal lamination regardless of treatment status. The loss of retinal cysts in this XLRS KO model has been previously described [26, 30, 32]. However, there were several intravitreal and subretinal AAV-treated eyes that had improvement in cysts and improved central macular thickness two weeks post-injection in only the treated eye, whereas all untreated *Rs1*-KO mice had essentially symmetric appearance between the eyes over the same time frame.

CAIs are used clinically to improve retinal cystic cavities [7, 10, 11, 13, 14, 33], and studies by Pennesi, et al. and Scruggs et al. have found that CAI treatment in XLRS patients improve visual acuity, often *without* improvement in retinal cyst volume [10, 32]. Zhour, et al. found that topical CAIs failed to improve retinal schisis in an *Rs1*-KO mouse model [32], but CAI-related functional studies have not been performed to date in this model. Thus, this study also tested the hypothesis that CAIs would improve or lengthen the time of effect in mice treated with RS1 subretinal gene therapy. Our ERG data and swim data in mice demonstrated no improvement in retinal function with Azopt^®^ treatment alone compared to untreated eyes. The mechanism and functionality of CAI treatment in humans with XLRS may be different than in mice.

The visually guided swim assay is a test of visual function, akin to the gold standard multiluminance mobility test used in human gene therapy trials to assess treatment effects [34]. We have demonstrated that in mice the swim assay correlates with visual acuity rather than memory and with loss of photoreceptors over time in mouse models of retinal disorders. The current study demonstrated for the first time that the dark-adapted assay provided the most meaningful comparisons across the mouse treatment groups in functional vision. Subretinal gene therapy was the only treatment that led to significant improvement in functional vision on the swim assay. There were no detectable differences in light-adapted vision between groups. In the dark, subretinal gene therapy significantly improved (i.e., decreased) the time to platform. Such functional improvements hold promise for similar advancements in affected patients.

Our data suggests that with this novel vector, the risks of subretinal injection in select patients with XLRS may be warranted due to better rescue of the retina. However, additional studies of retinal penetration using this vector are warranted. Further, subretinal bleb formation in XLRS patients has yet to be studied, and the retinal fragility, including frequent bullous schisis, of these patients must be considered to minimize risk of retinal detachment during surgery.

It is promising that gene therapy (e.g., voretigene neparvovec-rzyl) is already commercially available for subretinal injection for *RPE65-associated* Leber’s congenital amaurosis (LCA) [34], and other retinal dystrophies, such as choroideremia, achromatopsia and retinitis pigmentosa are being treated with subretinal gene therapy in phase I/II clinical trials, despite also having retinal fragility [35–38]. Suprachoroidal delivery might be an option in the future for XLRS as it avoids disturbing the retina [39, 40], and serial intravitreal or subretinal injections have yet to be studied in the mouse model or in patients. These approaches require further study.

AAV2/4 has known tropism for outer retinal layers [17], making it an excellent candidate for XLRS therapy. This is the first study to demonstrate that 1) AAV2/4 gene therapy is safe and effective in an animal model, 2) topical CAI therapy does not potentiate gene therapy in *Rs1-KO* mice, and 3) the visually guided swim assay can be used to compare treatment effects on vision function in a mouse model of XLRS. In this study, intravitreal and subretinal injections in the *Rs1-KO* mouse using the AAV2/4 vector increased the expression of RS1 in the retina, but in differing layers. Intravitreal gene therapy primarily transduced the inner retina whereas subretinal injections of AAV2/4-*RS1* increased expression in the outer retinal photoreceptors. Although both routes demonstrated some rescue, the subretinal route was more efficacious with significant improvement in ERG amplitudes and swim outcomes.

## Supporting information

S1 Video Visually guided swim assay in the dark. Three separate videos are compiled: wild type mouse, an untreated *Rs1*-KO mouse, and a *Rs1*-KO mouse treated subretinally with gene therapy. These videos were taken at postnatal month 6.
(MP4)

S1 Fig Vector map of AAV2/4-*RS1*. (TIF)

S2 Fig Localization of subretinal bleb formation and subsequent transduction in wild-type mice. **(A-C**) Representative merged image (**A**) of a color fundus image (**B**) and *in vivo* imaging of green fluorescent protein (GFP) fluorescence with blue light (**C**) 10 days after subretinal delivery of AAV2/4-*GFP* in a wild-type (WT) mouse. (**D**) Representative optical coherence tomography (OCT) image demonstrating a self-sealing retinal hole (white asterisk) and few opacities in vitreous after intravitreal administration of AAV2/4-*GFP* in a WT mouse. Some eyes receiving intravitreal gene therapy also had a very small subretinal bleb (white arrow, **D**). (**E)** OCT demonstrating a small subretinal bleb (white arrow, **E**).
(JPG)

S3 Fig ERG amplitudes over time with and without treatment with AAV2/4-*RS1*. Dark-adapted (DA) ERG and light-adapted (LA) ERG representative waveforms for a representative *Rs1-KO* mouse with subretinal AAV-treated eye and untreated eye at postnatal months 5.5 and 11. DA and LA ERGs for a WT were performed for comparison purposes at similar timepoints. For each tracing, time is shown in milliseconds (ms; x-axis) and amplitude is shown in microvolts (μv; y-axis). The b-wave amplitude for each waveform is noted by an asterisk (*). AAV, adeno-associated virus; DA, dark-adapted; ERG, electroretinography; LA, light-adapted; RS1, retinoschisin; WT, wild-type.
(JPG)

S4 Fig Full field ERG amplitudes improve with subretinal AAV2/4-*RS1* treatment. Dark-adapted (DA) ERG at 0.01, 0.1 and 1.0 cd.s/m^2^ and light-adapted (LA) ERG at 3.0 cd.s/m^2^ and 5 Hz flicker were performed on 12 *Rs1-KO* mice at postnatal months 9–10. All mice had subretinal AAV-treated right eyes (OD) and untreated left eyes (OS). Each color represents a different mouse with 10 observations (5 ERG intensities (x-axis) for 2 eyes). The log of the b wave amplitude is shown in log(microvolts) on the y-axis. DA, dark-adapted; ERG, electroretinography; KO, knockout; LA, light-adapted; OD, treated right eyes; OS, untreated left eyes; RS1, retinoschisin; WT, wild-type.
(JPG)
