# Supplementary figures and images for: AAV2/4-RS1 gene therapy in the retinoschisin knockout mouse model of X-linked retinoschisis
Source: PLoS One. 2022 Dec 7;17(12):e0276298. doi: 10.1371/journal.pone.0276298 (PMC9728878; doi:10.1371/journal.pone.0276298)

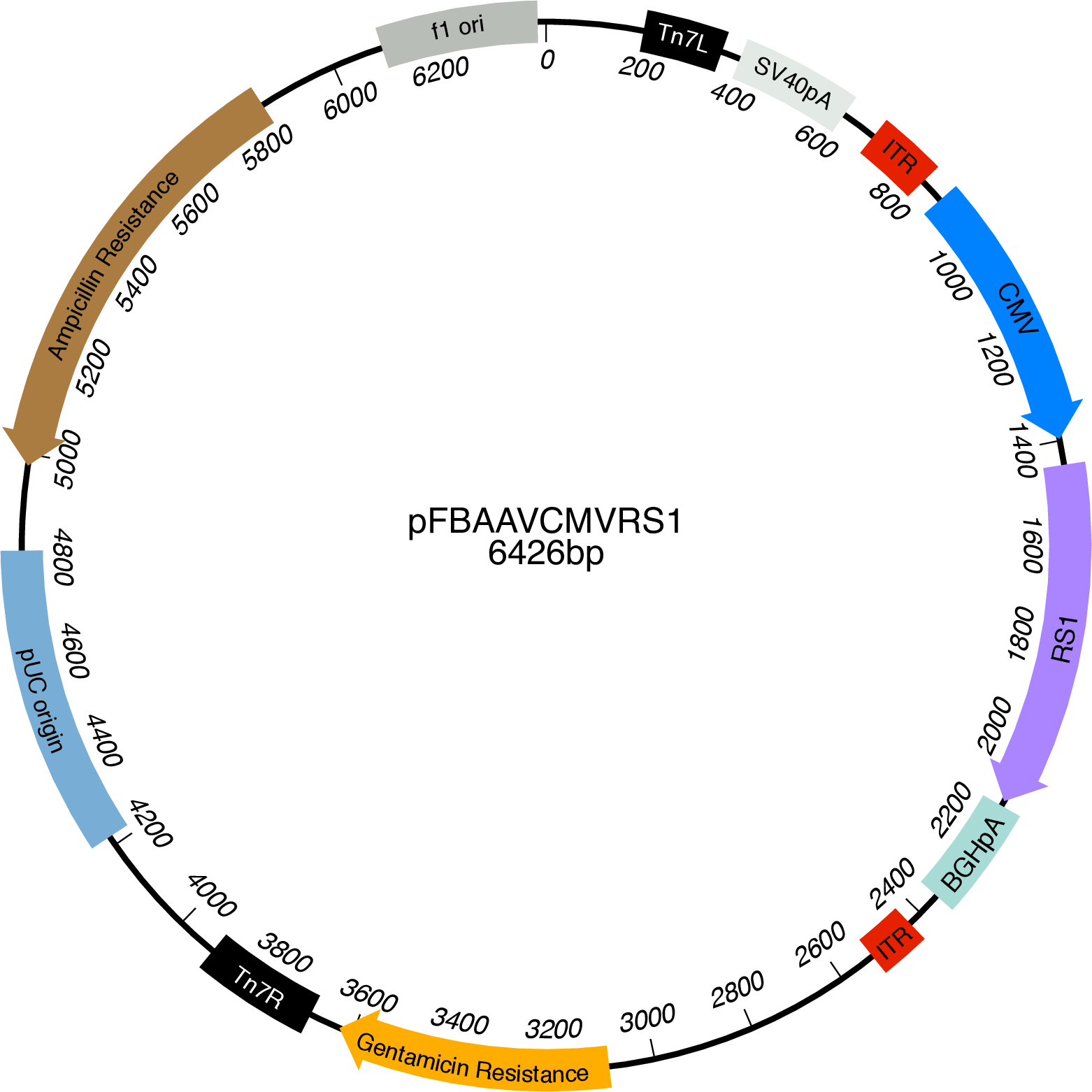

Supplement: S1 Fig — (TIF) [file pone.0276298.s002.tif]

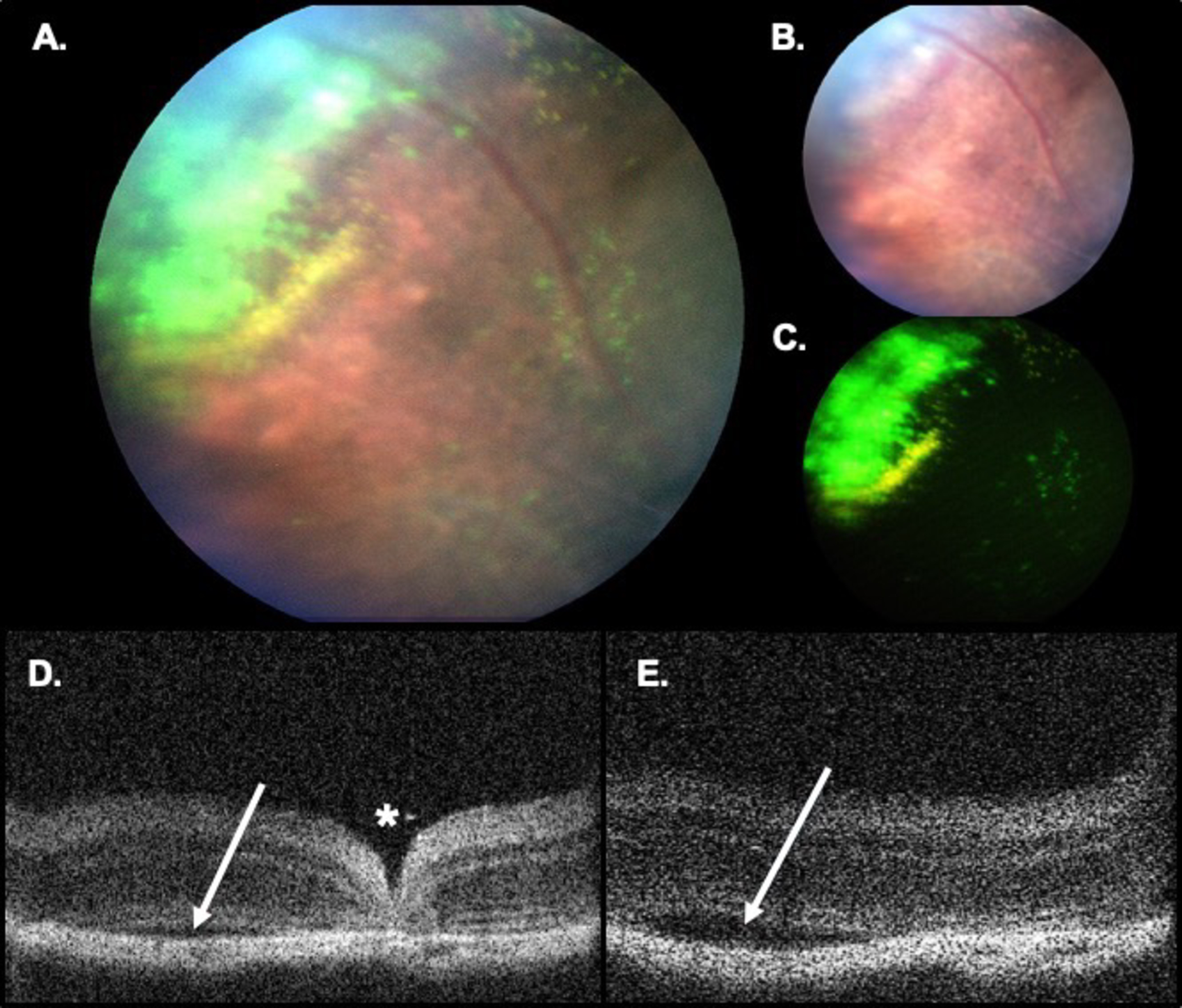

Supplement: S2 Fig — (A-C) Representative merged image (A) of a color fundus image (B) and in vivo imaging of green fluorescent protein (GFP) fluorescence with blue light (C) 10 days after subretinal delivery of AAV2/4-GFP in a wild-type (WT) mouse. (D) Representative optical coherence tomography (OCT) image demonstrating a self-sealing retinal hole (white asterisk) and few opacities in vitreous after intravitreal administration of AAV2/4-GFP in a WT mouse. Some eyes receiving intravitreal gene therapy also had a very small subretinal bleb (white arrow, D). (E) OCT demonstrating a small subretinal bleb (white arrow, E). (JPG) [file pone.0276298.s003.jpg]

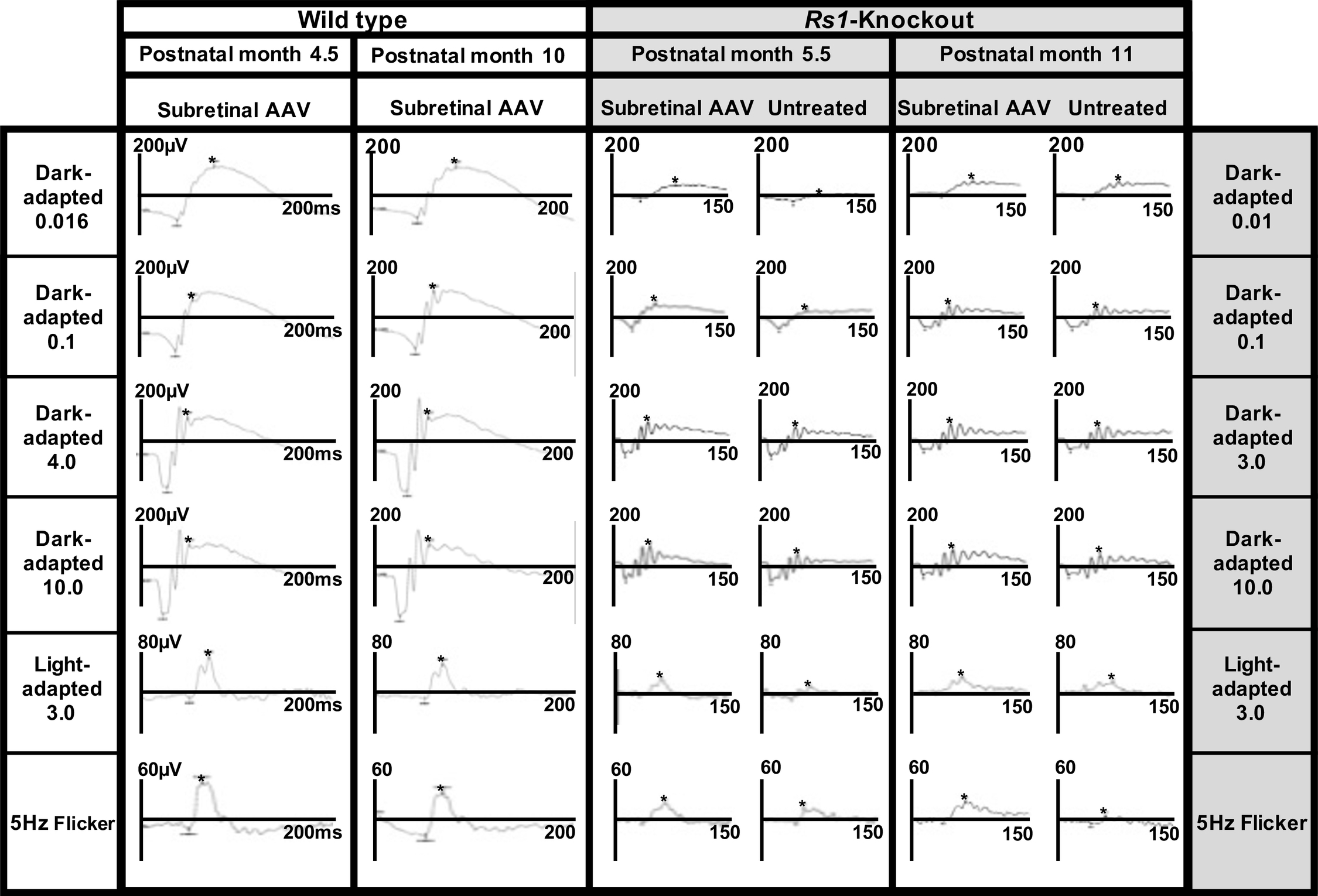

Supplement: S3 Fig — Dark-adapted (DA) ERG and light-adapted (LA) ERG representative waveforms for a representative Rs1-KO mouse with subretinal AAV-treated eye and untreated eye at postnatal months 5.5 and 11. DA and LA ERGs for a WT were performed for comparison purposes at similar timepoints. For each tracing, time is shown in milliseconds (ms; x-axis) and amplitude is shown in microvolts (μv; y-axis). The b-wave amplitude for each waveform is noted by an asterisk (*). AAV, adeno-associated virus; DA, dark-adapted; ERG, electroretinography; LA, light-adapted; RS1, retinoschisin; WT, wild-type. (JPG) [file pone.0276298.s004.jpg]

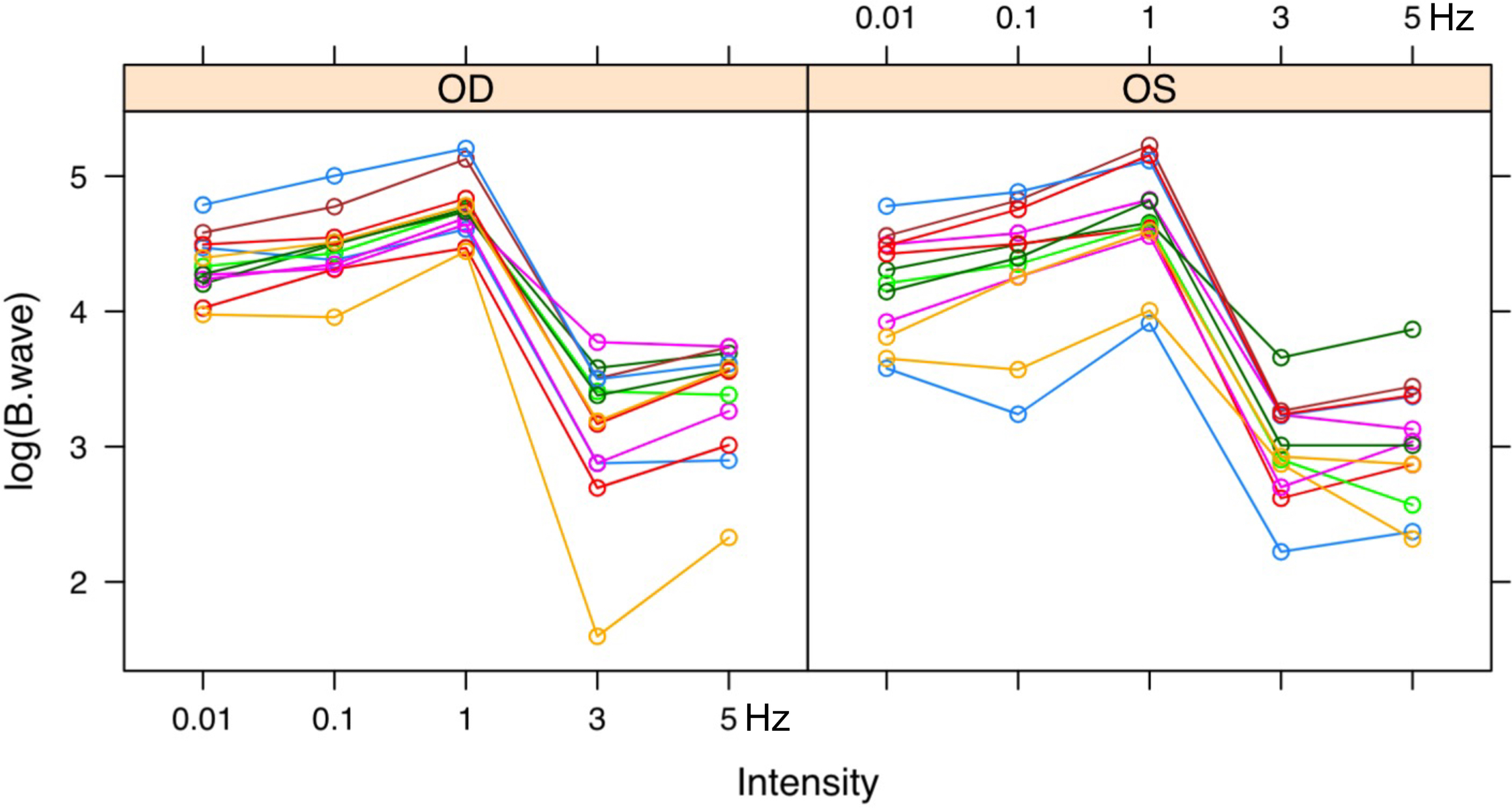

Supplement: S4 Fig — Dark-adapted (DA) ERG at 0.01, 0.1 and 1.0 cd.s/m2 and light-adapted (LA) ERG at 3.0 cd.s/m2 and 5 Hz flicker were performed on 12 Rs1-KO mice at postnatal months 9–10. All mice had subretinal AAV-treated right eyes (OD) and untreated left eyes (OS). Each color represents a different mouse with 10 observations (5 ERG intensities (x-axis) for 2 eyes). The log of the b wave amplitude is shown in log(microvolts) on the y-axis. DA, dark-adapted; ERG, electroretinography; KO, knockout; LA, light-adapted; OD, treated right eyes; OS, untreated left eyes; RS1, retinoschisin; WT, wild-type. (JPG) [file pone.0276298.s005.jpg]
